# Effectiveness of postoperative radiotherapy after radical cystectomy for locally advanced bladder cancer

**DOI:** 10.1002/cam4.2102

**Published:** 2019-05-22

**Authors:** Benjamin W. Fischer‐Valuck, Jeff M. Michalski, Nandita Mitra, John P. Christodouleas, Todd A. DeWees, Eric Kim, Zachary L. Smith, Gerald L. Andriole, Vivek Arora, Arnold Bullock, Ruben Carmona, Robert S. Figenshau, Robert L. Grubb, Thomas J. Guzzo, Eric M. Knoche, S. Bruce Malkowicz, Ronac Mamtani, Russell K. Pachynski, Bruce J. Roth, Mohamed S. Zaghloul, Hiram A. Gay, Brian C. Baumann

**Affiliations:** ^1^ Department of Radiation Oncology Washington University in St. Louis St. Louis Missouri; ^2^ Department of Radiation Oncology Emory University, Winship Cancer Institute Atlanta Georgia; ^3^ Department of Biostatistics, Epidemiology and Informatics University of Pennsylvania Philadelphia Pennsylvania; ^4^ Department of Radiation Oncology University of Pennsylvania Philadelphia Pennsylvania; ^5^ Mayo Clinic, Division of Biomedical Statistics and Informatics Scottsdale Arizona; ^6^ Department of Urology Washington University in St. Louis St. Louis Missouri; ^7^ Department of Medical Oncology Washington University in St. Louis St. Louis Missouri; ^8^ Department of Urology University of Pennsylvania Philadelphia Pennsylvania; ^9^ Department of Medical Oncology University of Pennsylvania Philadelphia Pennsylvania; ^10^ National Cancer Institute, Cairo University Cairo Egypt

**Keywords:** adjuvant radiation therapy, bladder cancer, PORT

## Abstract

**Background:**

Local‐regional failure (LF) for locally advanced bladder cancer (LABC) after radical cystectomy (RC) is common even with chemotherapy and is associated with high morbidity/mortality. Postoperative radiotherapy (PORT) can reduce LF and may enhance overall survival (OS) but has no defined role. We hypothesized that the addition of PORT would improve OS in LABC in a large nationwide oncology database.

**Methods:**

We identified ≥ pT3pN0‐3M0 LABC patients in the National Cancer Database diagnosed 2004‐2014 who underwent RC ± PORT. OS was calculated using Kaplan‐Meier and Cox proportional hazards regression modeling was used to identify predictors of OS. Propensity matching was performed to match RC patients who received PORT vs those who did not.

**Results:**

15,124 RC patients were identified with 512 (3.3%) receiving PORT. Median OS was 20.0 months (95% CI, 18.2‐21.8) for PORT vs 20.8 months (95% CI, 20.3‐21.3) for no PORT (*P* = 0.178). In multivariable analysis, PORT was independently associated with improved OS: hazard ratio 0.87 (95% CI, 0.78‐0.97); *P* = 0.008. A one‐to‐three propensity match yielded 1,858 patients (24.9% receiving PORT and 75.1% without). In the propensity‐matched cohort, median OS was 19.8 months (95% CI, 18.0‐21.6) for PORT vs 16.9 months (95% CI, 15.6‐18.1) for no PORT (*P* = 0.030). In the propensity‐matched cohort of urothelial carcinoma patients (N = 1,460), PORT was associated with improved OS for pT4, pN+, and positive margins (*P* < 0.01 all).

**Conclusion:**

In this observational cohort, PORT was associated with improved OS in LABC. While the data should be interpreted cautiously, these results lend support to the use of PORT in selected patients with LABC, regardless of histology. Prospective trials of PORT are warranted.

## 1 INTRODUCTION

Local‐regional failure (LF) for locally advanced bladder cancer (LABC) after radical cystectomy (RC) is common, and is associated with high morbidity and mortality.^1^, ^2^, ^3^ Adjuvant chemotherapy has not been shown in randomized prospective trials to reduce the risk of LFs,^1^, ^4^ and salvage strategies after LF are rarely successful.^2^, ^5^, ^6^ Postoperative radiotherapy (PORT) has been shown to significantly reduce local failures and may enhance survival.^7^, ^8^ A recently published phase II randomized trial in Egypt of patients with LABC status post RC and pelvic lymph node dissection with negative margins reported significantly improved local control with the addition of PORT vs adjuvant chemotherapy alone, with 2‐year local control of 96% for sequential chemotherapy plus PORT vs 69% for chemotherapy alone (*P* < 0.01).^8^ Disease‐free survival and overall survival (OS) were improved with the addition of PORT but the study was not powered for those endpoints. While only 53% of the patients had urothelial carcinoma, outcomes did not differ based on histology.

Interest in PORT after RC has increased in Europe and North America, and researchers have identified an externally validated risk stratification for selecting patients at highest risk for local failure who are most likely to benefit from PORT and have mapped the patterns of failure in the pelvis to design consensus target volumes.^1^, ^9^, ^10^, ^11^, ^12^, ^13^ An NRG randomized phase II trial of PORT vs no PORT (NRG‐GU001) opened in 2015 in the US and Canada but closed early due to poor accrual. Other trials of PORT in Europe, India, and Egypt have opened, but are not powered for an OS endpoint.^14^ It is unlikely that a randomized trial of sufficient size can be conducted in the West to assess whether PORT improves OS, and large retrospective series are lacking. The purpose of this study is to investigate whether the addition of PORT improved OS using the National Cancer Database (NCDB), a database of sufficient size to potentially answer the question. We hypothesized that the addition of PORT would improve overall survival in patients with LABC.

## 2 MATERIALS AND METHODS

### 2.1 Study population

The NCDB Participant User File for bladder tumors was reviewed to identify all patients 18‐90 years old diagnosed with bladder cancer from 2004 to 2014. Data from approximately 70% of the patients diagnosed at Commission on Cancer‐accredited cancer centers is incorporated and includes patient, tumor, and treatment characteristics. The Participant User File contains de‐identified patient and center information and was exempt from Institutional Review Board review.

From this dataset, we selected a cohort of patients who would have been eligible for the NRG‐GU001 study as follows. All patients included received cystectomy as defined by cystectomy, RC, or more advanced surgical procedure (ie, exenteration). Only patients with pT3‐4,N0‐3,M0 disease, known surgical margin status, nonsmall cell and nonlymphoma histology, and known chemotherapy details were included. Additionally, those patients who died within 30 days of surgery or did not have follow‐up information were excluded. Lastly, patients with more favorable disease characteristics (pT3a,N0 and ≥ 10 LN dissected, and negative surgical margins) were excluded as these patients have been shown to have lower risk of LF and were excluded from NRG‐GU001.^10^ Patients were classified into two cohorts: postoperative radiotherapy (PORT) vs no PORT. Patients included in the PORT cohort received postoperative external beam radiotherapy to the pelvis/cystectomy bed within 1 year of surgery to a total dose of ≥ 40 Gy. Patients receiving palliative pelvic radiation therapy as coded by the NCDB were excluded. Patients who died within 30 days of surgery were excluded. Patient CONSORT diagram detailing complete inclusion criteria is found in Figure S1.

Patient characteristics for analysis included: age, sex, race, Charlson‐Deyo comorbidity index (CCI), treatment facility type, primary insurance status, histology, pathologic T‐stage, pathologic N‐stage, number of regional nodes examined, surgical margin status, receipt of chemotherapy (both neoadjuvant and adjuvant), and receipt of radiotherapy. The primary endpoint was overall survival.

### 2.2 Statistical analysis

The chi‐squared test was used to compare categorical demographic and patient characteristics between the two treatment groups. The Student's *t*test was used to compare continuous variables between groups. Overall survival was calculated from diagnosis until death, censoring at last follow‐up for patients who were alive. The Kaplan‐Meier method was used to estimate overall survival probabilities. Univariable (UVA) and multivariable analysis (MVA) logistic regression modeling were used to identify predictors of receiving adjuvant radiotherapy and are reported as odds ratios. UVA and MVA Cox proportional hazard modeling were used to identify factors associated with overall survival and are reported as hazard ratios (HR) with corresponding 95% confidence intervals. The MVA models were created by including all covariates and then removing each covariate with a *P* value > 0.2 in a step‐wise method. Categorical covariates were included in the final model if the covariate levels in comparison with the reference group had a *P* value < 0.1.^15^ To confirm appropriate selection of predictive variables entered into multivariable analysis, a stepwise regression was utilized. Proportional hazards assumptions were tested using Schoenfeld residuals tests and were not violated. *P* < 0.05 was considered significant. All were two‐sided.

Since observational studies are susceptible to unmeasured confounding, we conducted a regression‐based sensitivity analysis in which we evaluated the sensitivity of our Cox HR to the presence of a binary confounder (such as patient functional status which was not available in our dataset). We varied the prevalence and strength of the unmeasured confounder to assess whether our primary findings would be altered if in fact we could have adjusted for the unmeasured confounder.^16^

A secondary propensity score (PS) matched analysis was conducted to better potentially account for differences in baseline patient characteristics between the PORT and no PORT groups. Matching was performed based on patient characteristics and disease factors that included: age, sex, race, CCI, facility type, insurance status, histology, pathologic T‐stage, pathologic N‐stage, margin status, number of nodes examined, and chemotherapy treatment information including neoadjuvant vs adjuvant. One‐to‐three matching using nearest‐neighbor algorithm assuming independent observations and fixed weights was performed. Caliper width was narrowed in a stepwise fashion until the covariate distributions were balanced after matching.^17^ A caliper width of 0.2 was used in subsequent analyses. Balancing of groups after PS matching was verified using the χ^2^ test for categorical variables and the *t*test for continuous variables as well as comparing standardized differences of baseline covariates between the PORT and no PORT groups. After matching, a matched‐sample UVA Cox regression model was applied to the matched groups to estimate the effect of treatment on survival.^18^ Forest plots were generated after PS matching using UVA Cox regression to analyze the subgroup interactions. SPSS Statistics v.23 (IBM Corporation; Armonk, NY) was used for all statistical analyses.

## 3 RESULTS

### 3.1 Demographics and factors associated with receipt of PORT

Of the 484,367 patients diagnosed with bladder cancer from 2004 to 2014 in the NCDB, we identified 15,124 patients who met inclusion criteria (Figure S1). Median follow‐up was 18.8 months (25‐75th quartile: 9.8‐39.0 months). Five hundred and twelve (3.3%) of the patients received PORT. Median time from surgery to PORT was 110 days [25‐75th quartile: 52‐188 days]. Median radiation dose was 50.4 Gy [25‐75th quartile: 45‐55.80 Gy]. Median age of patients receiving PORT was 65 years vs 69 years for no PORT (*P* < 0.0001). Baseline patient characteristics are listed in Table 1. Multivariable logistic regression showed that cofactors associated with increased likelihood for PORT included: female gender, nonurothelial histology, pathologic T4 stage, positive surgical margins, and receipt of chemotherapy (Table 2).

**Table 1:** Demographics and clinical characteristics

	Number of Patients		*P*‐Value
	No PORT	PORT	
N	14,612 (96.7%)	512 (3.3%)	
Age, years			**<0.0001**
Mean	68.0	64.6	
SD	10.6	10.8	
Median	69	65	
Range	22‐90	32‐90	
Sex			
Male	10,583 (72.4%)	319 (62.3%)	**<0.0001**
Female	4,029 (27.6%)	193 (37.7%)	
Race			
White	13,240 (90.6%)	452 (88.3%)	0.074
Other	1,372 (9.4%)	60 (11.7%)	
Charlson‐Deyo Comorbidity:			
0	10,228 (70.0%)	375 (73.2%)	0.247
1	3,328 (22.8%)	101 (19.7%)	
≥2	1,056 (7.2%)	36 (7.0%)	
Facility Type			
Academic/Research Program	7,248 (49.6%)	157 (30.7%)	**<0.0001**
Other	7,263 (49.7%)	346 (67.6%)	
Unknown	101 (0.7%)	9 (1.8%)	
Insurance Status			
Private	4,470 (30.6%)	185 (36.1%)	**0.004**
Other	10,142 (69.4%)	327 (63.9%)	
Histology			
Urothelial	12,972 (88.8%)	389 (76.0%)	**<0.0001**
Squamous	860 (5.9%)	69 (13.5%)	
Adeno	271 (1.9%)	22 (4.3%)	
Other (excluding small cell/lymphoma)	509 (3.5%)	32 (6.3%)	
Pathologic T‐stage			
T3	9,729 (66.6%)	219 (42.8%)	**<0.0001**
T4	4,883 (33.4%)	293 (57.2%)	
Positive Lymph Nodes			
No	7,678 (52.5%)	258 (50.4%)	0.345
Yes	6,934 (47.5%)	254 (49.6%)	
Number of Regional Lymph Nodes Examined			
Mean	12.9	10.3	0.946
SD	12.3	10.2	
Median	9.0	8.0	
Range	0‐90	0‐62	
Positive surgical margins			
No	11,707 (80.1%)	249 (48.6%)	**<0.0001**
Yes	2,905 (19.9%)	263 (51.4%)	
Chemotherapy			
None	8,329 (57.0%)	119 (23.2%)	**<0.0001**
Single‐agent	474 (3.2%)	98 (19.1%)	
Multi‐agent	5,382 (36.8%)	263 (51.4%)	
Number of agents unknown	428 (2.9%)	32 (6.3%)	
Chemotherapy Sequence			**<0.0001**
None	8,201 (56.1%)	118 (23.0%)	
Neoadjuvant	1,405 (9.6%)	48 (9.4%)	
Adjuvant	4,311 (29.5%)	311 (60.7%)	
Both	415 (2.8%)	23 (4.5%)	
Unknown	280 (1.9%)	12 (2.3%)	

**Table 2:** Univariable and multivariable logistic regression for receipt of adjuvant RT

	Univariate		Multivariate	
	Odds ratio	*P*‐Value	Odds ratio	*P*‐Value
Age				
Years	**0.97 (0.96‐0.98)**	**<0.0001**	**0.98 (0.97‐0.99)**	**0.003**
Sex				
Male	Reference Group		Reference Group	
Female	**1.57 (1.31‐1.89)**	**<0.0001**	**1.47 (1.20‐1.80)**	**<0.0001**
Race				
White	Reference Group		Reference Group	
Other	0.79 (0.60‐1.04)	0.095	0.82 (0.61‐1.11)	0.201
Charlson‐Deyo Comorbidity:				
0	Reference Group		Reference Group	
1	0.83 (0.66‐1.04)	0.109	0.93 (0.73‐1.18)	0.456
≥2	0.93 (0.63‐1.29)	0.558	1.12 (0.77‐1.63)	0.434
Facility Type				
Academic/Research Program	**0.45 (0.37‐0.55)**	**<0.0001**	**0.48 (0.39‐0.60)**	**<0.0001**
Other	Reference Group		Reference Group	
Unknown	1.92 (0.96‐3.82)	0.075	0.91 (0.41‐2.03)	0.816
Insurance Status				
Private Insurance	**1.29 (1.07‐1.56)**	**0.008**	1.03 (0.82‐1.29)	0.790
Other	Reference Group		Reference Group	
Histology				
Urothelial	Reference Group		Reference Group	
Squamous	**2.70 (2.07‐3.53)**	**<0.0001**	**2.65 (1.96‐3.60)**	**<0.0001**
Adeno	**2.51 (1.58‐4.00)**	**<0.0001**	**2.07 (1.24‐3.44)**	**0.005**
Other (excluding small cell/lymphoma)	**2.15 (1.49‐3.12)**	**<0.0001**	**1.59 (1.06‐2.39)**	**0.025**
Pathologic T‐stage				
T3	Reference Group		Reference Group	
T4	**2.84 (2.37‐3.40)**	**<0.0001**	**2.04 (1.67‐2.49)**	**<0.0001**
Positive Lymph Nodes				
No	Reference Group		Reference Group	
Yes	1.15 (0.96‐1.38)	0.125	0.90 (0.74‐1.10)	0.290
Number of Regional Lymph Nodes Examined	**0.98 (0.97‐0.99)**	**<0.0001**	**0.98 (0.97‐0.99)**	**<0.0001**
Positive Surgical Margins				
No	Reference Group		Reference Group	
Yes	**4.50 (3.75‐5.39)**	**<0.0001**	**3.31 (2.71‐4.03)**	**<0.0001**
Chemotherapy				
None	Reference Group		Reference Group	
Single‐agent	**5.19 (3.45‐7.80)**	**<0.0001**	**4.66 (3.03‐7.17)**	**<0.0001**
Multi‐agent	**14.12 (10.6‐18.8)**	**<0.0001**	**11.66 (8.52‐15.9)**	**<0.0001**
Number of agents unknown	**3.44 (2.76‐4.29)**	**<0.0001**	**3.27 (2.57‐4.17)**	**<0.0001**

### 3.2 Survival analysis

**Figure 1:**
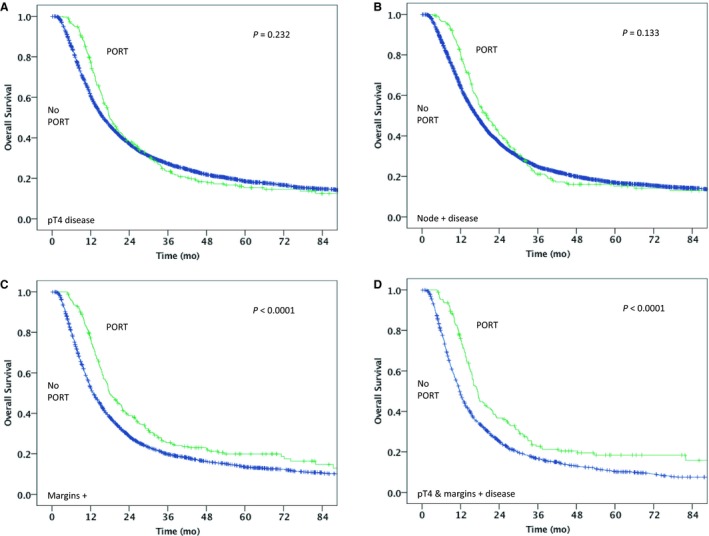
Kaplan‐Meier overall survival curves for PORT vs no PORT in: (A) pathologic T4 (pT4) disease; (B) node positive disease; (C) positive surgical margins; (D) both pT4 disease and positive surgical margins. PORT, postoperative radiotherapy. Green line = PORT, Blue line = No PORT

The median follow‐up for patients receiving PORT was 18.6 months vs 18.8 months in the no PORT group. The median OS was 20.0 months (95% CI, 18.2‐21.8) for the PORT group vs 20.8 months (95% CI, 20.3‐21.3) for the group that did not receive PORT (*P* = 0.178) (Figure S2). For patients with pT4 disease, the median OS was 17.9 months (95% CI, 16.3‐19.6) for PORT vs 15.9 months (95% CI, 15.2‐16.5) for no PORT (*P* = 0.232) (Figure 1A). Patients with node‐positive disease had a median OS of 20.1 months (95% CI, 17.1‐23.2) for PORT vs 17.0 (95% CI, 16.5‐17.5) for no PORT (*P* = 0.133) (Figure 1B). For patients with positive surgical margins, the median OS was 17.9 months (95% CI, 15.6‐20.1) for PORT vs 12.8 months (95% CI, 12.2‐13.4) for no PORT (*P* < 0.0001) (Figure 1C). For patients with both pT4 disease and positive surgical margins, the median OS was 17.3 months (95% CI, 15.8‐18.8) for PORT vs 11.7 months (95% CI, 11.1‐12.2) for no PORT (*P* < 0.0001) (Figure 1D). In multivariable analysis, PORT was independently associated with an improved OS (HR: 0.87 [95% CI, 0.78‐0.97]; *P* = 0.008) (Table 3).

**Table 3:** Univariable and multivariable Cox regression for overall survival

	Univariable		Multivariable	
	Hazard ratio	*P*‐Value	Hazard ratio	*P*‐Value
Postoperative RT (PORT)	1.09 (0.98‐1.21)	0.101	0.87 (0.78‐0.97)	**0.008**
Age				
Years	1.01 (1.01‐1.02)	**<0.0001**	1.01 (1.01‐1.01)	**<0.0001**
Sex				
Male	Reference Group		Reference Group	
Female	1.05 (1.01‐1.10)	**0.013**	1.03 (0.99‐1.08)	0.124
Race				
White	Reference Group		Reference Group	
Other	0.94 (0.89‐1.01)	0.077	0.92 (0.86‐0.98)	**0.009**
Charlson‐Deyo Comorbidity				
0	Reference Group		Reference Group	
1	1.21 (1.16‐1.27)	**<0.0001**	1.17 (1.12‐1.22)	**<0.0001**
≥2	1.35 (1.26‐1.45)	**<0.0001**	1.28 (1.19‐1.38)	**<0.0001**
Facility Type				
Academic/Research Program	0.95 (0.92‐0.99)	**0.013**	0.96 (0.93‐1.00)	0.060
Other	Reference Group		Reference Group	
Unknown	0.86 (0.68‐1.08)	0.199	1.24 (0.98‐1.58)	0.078
Insurance Status				
Private Insurance	0.78 (0.75‐0.81)	**<0.0001**	0.91 (0.87‐0.96)	**<0.0001**
Other	Reference Group		Reference Group	
Histology				
Urothelial	Reference Group		Reference Group	
Squamous	1.12 (1.07‐1.25)	**<0.0001**	1.18 (1.09‐1.28)	**<0.0001**
Adeno	0.89 (0.77‐1.02)	0.090	0.82 (0.72‐0.95)	**0.008**
Other (excluding small cell/lymphoma)	1.22 (1.10‐1.34)	**<0.0001**	1.12 (1.01‐1.24)	**0.025**
Pathologic T‐stage				
T3	Reference Group		Reference Group	
T3a	1.04 (0.97‐1.12)	0.230	0.98 (0.91‐1.05)	0.616
T3b	1.18 (1.10‐1.27)	**<0.0001**	1.21 (1.13‐1.31)	**<0.0001**
T4	1.52 (1.37‐1.67)	**<0.0001**	1.37 (1.27‐1.52)	**<0.0001**
T4a	1.44 (1.34‐1.54)	**<0.0001**	1.36 (1.27‐1.48)	**<0.0001**
T4b	2.21 (1.98‐2.47)	**<0.0001**	2.02 (1.80‐2.27)	**<0.0001**
Positive Lymph Nodes				
No	Reference Group		Reference Group	
Yes	1.45 (1.45‐1.57)	**<0.0001**	1.79 (1.72‐1.87)	**<0.0001**
Number of Regional Lymph Nodes Examined	0.99 (0.99‐0.99)	**<0.0001**	0.99 (0.99‐0.99)	**<0.0001**
Positive surgical margins				
No	Reference Group		Reference Group	
Yes	1.68 (1.60‐1.75)	**<0.0001**	1.51 (1.44‐1.59)	**<0.0001**
Chemotherapy				
None	Reference Group		Reference Group	
Single‐agent	0.95 (0.86‐1.05)	0.338	0.74 (0.67‐0.83)	**<0.0001**
Multi‐agent	0.78 (0.75‐0.81)	**<0.0001**	0.69 (0.66‐0.72)	**<0.0001**
Number of agents unknown	0.85 (0.76‐0.95)	**0.004**	0.83 (0.75‐0.92)	**<0.0001**

### 3.3 Sensitivity analysis

We performed a sensitivity analysis to assess the potential effect of unmeasured confounding on the primary outcome of overall survival. We chose patient functional status, which was not available to us in this study, although the analysis would apply to other unmeasured confounders, such as smoking status. Our sensitivity analysis showed that if there was an unmeasured confounder with a deleterious effect on OS with a HR of 1.25 and was 9% more common in the no PORT cohort, adjusting for it would not change the overall findings that PORT is associated with significantly improved OS (updated HR 0.90, 95% CI 0.80‐0.99). If the prevalence of the deleterious unmeasured confounder in the control group was much greater, for example 20% higher, PORT would no longer be statistically significant (HR 0.90, 95% CI 0.85‐1.04).

### 3.4 Matched analysis

**Figure 2:**
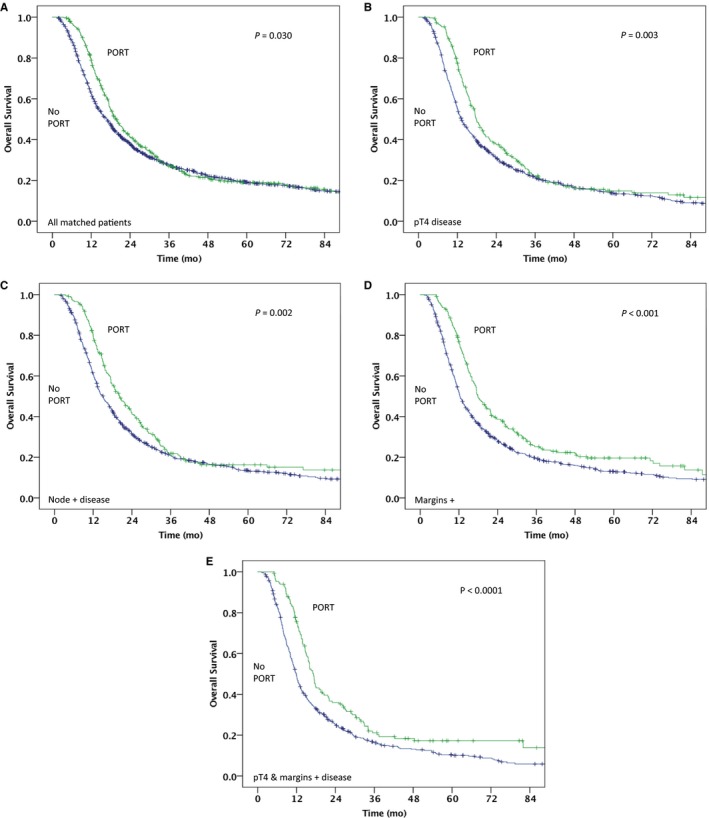
Kaplan‐Meier overall survival curves in the propensity score matched cohort for PORT vs no PORT in: (A) entire matched cohort; (B) matched pathologic T4 (pT4) disease; (C) matched node positive disease; (D) matched positive margins; (E) matched both pT4 disease and positive surgical margins. PORT, postoperative radiotherapy. Green line = PORT, Blue line = No PORT

**Figure 3:**
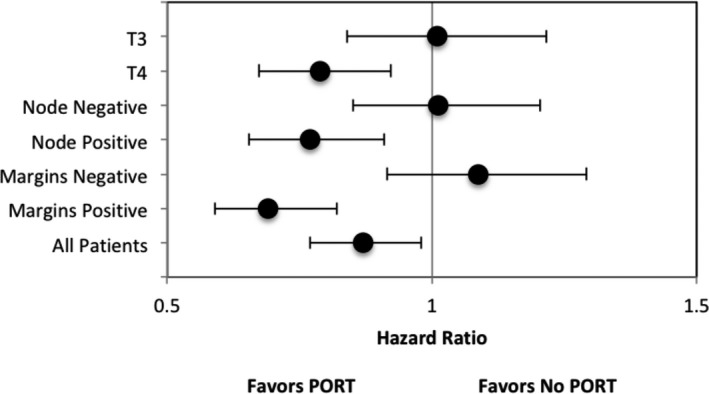
Forest plot of patient disease and treatment characteristics and association with overall survival

A one‐to‐three propensity match yielded a total of 1,858 patients (24.9% receiving PORT and 75.1% without receipt of PORT) (Table S1). In the propensity‐matched cohort, median OS was 19.8 months (95% CI, 18.0‐21.6) for the PORT group vs 16.9 months (95% CI, 15.6‐18.1) for the group that did not receive PORT (log‐rank *P* = 0.030, Wilcoxon *P* < 0.0001, Tarone‐Ware *P* < 0.0001) (Figure 2A). For patients with pT4 disease, the median OS was 17.9 months (95% CI, 16.2‐19.4) for PORT vs 13.2 months (95% CI, 12.2‐14.3) for no PORT (*P* = 0.003) (Figure 2B). For patients with node‐positive disease, the median OS was 20.2 months (95% CI, 17.4‐23.0) for PORT vs 15.1 (95% CI, 13.7‐16.4) for no PORT (*P* = 0.003) (Figure 2C). For patients with positive surgical margins, the median OS was 17.8 months (95% CI, 15.8‐19.8) for PORT vs 12.4 months (95% CI, 11.5‐13.2) for no PORT (*P* = 0.002) (Figure 2D). For patients with both pT4 disease and positive surgical margins, the median OS was 17.2 months (95% CI, 15.8‐18.6) for PORT vs 11.9 months (95% CI, 11.0‐12.7) for no PORT (*P* < 0.0001) (Figure 2E). PORT was independently associated with improved OS in the matched cohort (HR: 0.88 [95% CI, 0.77‐0.98]; *P* = 0.030). A forest plot of tumor and treatment characteristics and their association with OS is depicted in Figure 3.

### 3.5 Urothelial cohort

From the propensity‐matched cohort, 1,460 patients with urothelial histology were identified. Three hundred and fifty‐seven (24.5%) patients in this subgroup received PORT and 1,103 (75.5%) patients did not receive PORT. Other patient characteristics and receipt of chemotherapy in the urothelial cohort were well balanced (data not shown). Median OS was 20.2 months (95% CI, 18.2‐22.3) for the patients that received PORT compared to 17.2 months (95% CI, 15.8‐18.2) for no PORT (*P* = 0.099). For urothelial patients with pT4 disease, the median OS was 18.6 months (95% CI, 16.4‐20.8) for PORT vs 13.6 months (95% CI, 12.3‐14.8) for no PORT (*P* = 0.007) (Figure S3A). For patients with node‐positive disease, the median OS was 20.7 months (95% CI, 17.8‐23.6) for PORT vs 15.3 (95% CI, 13.8‐16.9) for no PORT (*P* = 0.011) (Figure S3B). For patients with positive surgical margins, the median OS was 18.3 months (95% CI, 15.9‐20.7) for PORT vs 12.9 months (95% CI, 11.6‐14.3) for no PORT (*P* = 0.002) (Figure S3C). For patients with both pT4 disease and positive surgical margins, the median OS was 17.4 months (95% CI, 15.7‐19.1) for PORT vs 11.9 months (95% CI, 10.9‐12.9) for no PORT (*P* = 0.002) (Figure S3D).

## 4 DISCUSSION

Local‐regional failure for LABC after RC is common. In both SWOG 8710 and Medical Research Council trials of RC with or without neoadjuvant chemotherapy, the 5‐year incidence of LFs for patients with ≥ pT3 urothelial carcinoma was over 30%.^1^, ^4^ It has been hypothesized that reducing LFs may lead to improved disease‐free and overall survival. From a surgical perspective, retrospective series have associated more extensive nodal dissections with improved survival outcomes, even in the absence of nodal metastasis, which suggests that removal of occult nodal disease may improve survival by decreasing LFs.^19^ Additionally, there is an evidence that local failure often precedes but uncommonly follows the development of distant metastasis, suggesting that local failure may seed distant disease.^20^ Even if LF did not reduce the risk of DM or affect survival, there is often considerable morbidity associated with LF and efforts to reduce LF may improve patient quality‐of‐life.^2^

Given the association between LF and development of distant disease, methods to improve local control may be warranted and may improve survival. While the addition of chemotherapy to RC improves overall survival, it has not been shown in randomized prospective trials to reduce the risk of LF.^1^, ^4^ As improvements in systemic therapy further reduce the risk of distant disease, treatments designed to reduce local recurrences will gain in importance to reduce the overall risk of relapse. Additionally, salvage strategies after LF are rarely successful with a median survival of approximately 9 months.^21^, ^22^ PORT for LABC thus offers an option to significantly improve local control, which may in turn improve survival, but the role of PORT has not been clearly defined.

Concerns for significant toxicity after PORT have been a major reason why adoption of this adjuvant therapy has been rather limited. Toxicity results using outdated radiotherapy techniques in the 1970s and 1980s did show relatively high rates of toxicity. However, the results of the Zaghloul et al trial of post‐operative radiotherapy plus adjuvant chemotherapy vs adjuvant chemotherapy alone after RC that used more modern three‐dimensional conformal radiotherapy reported low rates of late GI toxicity for PORT.^8^ In addition, a patterns of failure analysis by Baumann et al demonstrated that the cystectomy bed could be safely omitted for patients with negative margins, thus significantly reducing the amount of radiation dose to the central pelvis since only the pelvic side wall nodes would have to be covered to full dose.^9^ The radiation target volumes for PORT in patients with negative margins are routinely smaller than those commonly used for patients with prostate cancer receiving postoperative whole pelvis radiotherapy.

The use of PORT vs no PORT following RC in patients with LABC has not been evaluated in large, modern phase III trials powered to detect a benefit in overall survival. A previous randomized clinical trial of adjuvant RT vs observation conducted in the 1980s at the National Cancer Institute in Cairo, Egypt reported a significant improvement in both local control and disease‐free survival with PORT.^7^ In that study, 80% of the patients had squamous cell carcinoma and only 20% had urothelial carcinoma but the outcomes were equivalent independent of histology. That trial, which used older two‐dimensional RT techniques, established PORT as a standard adjuvant treatment for LABC in Egypt. A second randomized trial conducted at the NCI in Cairo compared sequential PORT and chemotherapy (n = 75) vs adjuvant chemotherapy alone (n = 45) in patients with LABC who had complete (R0) resections and again confirmed a significant benefit in local control.^8^ DFS and OS were improved but the study was not powered for those endpoints and the differences were not significant. In that trial, 53% of the patients had urothelial carcinoma, which may make these results more applicable to a western patient population. In an unplanned subset analysis of patients with urothelial carcinoma, the addition of PORT was also associated with a statistically significant improvement in local control.^8^

Interest in PORT has grown outside of Egypt and the Middle East, 23 and several trials of adjuvant RT have opened recently, including a cooperative group trial in France (GETUG) and single‐institution trials at Tata Memorial Hospital (Mumbai, India) and Ghent University (Ghent, Belgium).^14^, ^24^ The NRG Oncology cooperative group opened a trial in 2015 to evaluate the benefit of PORT for LABC that enrolled patients with pT3‐4 N0‐2 M0 bladder cancer after RC (NRG‐GU001). Unfortunately, the trial closed in 2017 due to poor accrual with insufficient patient numbers for analysis.

Given the closure of NRG‐GU001 and the challenges of accruing patients on trials of PORT in Europe and North America, it is unlikely that a phase III trial powered to detect an overall survival difference could be successfully completed in the near future. Therefore, we are limited to retrospective analyses of large population‐based databases. Fortunately, the selection criteria for patients who are most likely to benefit from PORT has been developed and externally validated and this information was incorporated in the selection criteria for NRG‐GU001.^1^, ^10^, ^25^, ^26^ In this study, we identified a patient population similar to the inclusion criteria for NRG‐GU001 and evaluated the role of PORT in this patient population.

We found that PORT was independently associated with an overall survival benefit on multivariable analysis. While important prognostic covariates such as age, T‐stage, positive margins, and receipt of chemotherapy were not balanced between the groups, after balancing these potential confounders with propensity matching, the association between improved OS and PORT became statistically stronger. Additionally, we found that the patient characteristics associated with the greatest overall survival benefit for PORT were pT4 disease, node positive disease, and positive surgical margins, characteristics which are associated with higher rates of local‐regional recurrence in the literature.^2^, ^22^, ^27^ We observed the same trends in the subgroup analysis of patients with urothelial histology with significant improvement in overall survival with the addition of PORT in pT4 disease, node positive disease, and positive surgical margins. This is the first study to report an overall survival benefit for PORT in LABC patients and lends further support to the change in the National Comprehensive Cancer Network (NCCN) guidelines that added PORT as a treatment option to consider for patients with LABC. It should be noted, however, that the patients included in this analysis were treated prior to the NCCN guidelines incorporating PORT and it may be that patients referred for PORT in this cohort had very poor prognostic factors, which would not be well‐captured in the NCDB (ie, grossly positive margins).

Although the strengths of our study include the large number of patients treated in the modern RT era, there are notable limitations. As an observational study, we were unable to control for all potential confounding factors that may influence the apparent survival benefit of PORT, though PS matching helps control for a large number of measured confounders. Key variables are not included in the NCDB, including information on cause of death and recurrence as well as radiation treatment volumes (ie, pelvis vs cystectomy bed alone). Detailed information on chemotherapeutic agents (ie, cisplatin vs noncisplatin based) used and their dosing are also not available in the NCDB. There is also a possible selection bias with respect to treatment assignment that cannot be fully adjusted for on a multivariable analysis and with matching. Patients receiving PORT may be representative of healthier patients who can tolerate additional therapy, or conversely, may be representative of patients with particularly advanced disease who were referred for a nonstandard adjuvant therapy. We conducted a sensitivity analysis to assess the robustness of our findings to unmeasured confounding to try to address these limitations. Lastly, in our study, the OS for patients may be worse than OS reported in clinical trials such as SWOG 8710 and other institutional retrospective series.^1^, ^14^ This difference may be related to inclusion of patients with comorbidities who would have been excluded from a clinical trial as well as heterogeneity in the radiation dose, treatment volumes, and chemotherapy administration.^28^, ^29^ Importantly, the lack of details on chemotherapeutic regimens used in this study (eg, chemotherapy agent(s), number of cycles administered, and doses) is a major limitation of the database.

To our knowledge, this is the largest study investigating the impact of PORT on OS in patients with LABC. Based on this retrospective analysis, PORT appears to be associated with improved OS and these findings lend support to the use of PORT. While not definitive, these results suggest that patients with LABC should be considered for PORT. The benefit for PORT appears to be particularly pronounced for pT4 disease, positive nodes, and/or positive margins. Phase 3 trials of PORT for patients with LABC are warranted.

## CONFLICT OF INTEREST

JC discloses part‐time employment at Elekta AB. The authors have no other conflict of interest.
