# Supplementary figures and images for: Effectiveness of postoperative radiotherapy after radical cystectomy for locally advanced bladder cancer
Source: Cancer Med. 2019 May 22;8(8):3698–709. doi: 10.1002/cam4.2102 (PMC6639450; doi:10.1002/cam4.2102)

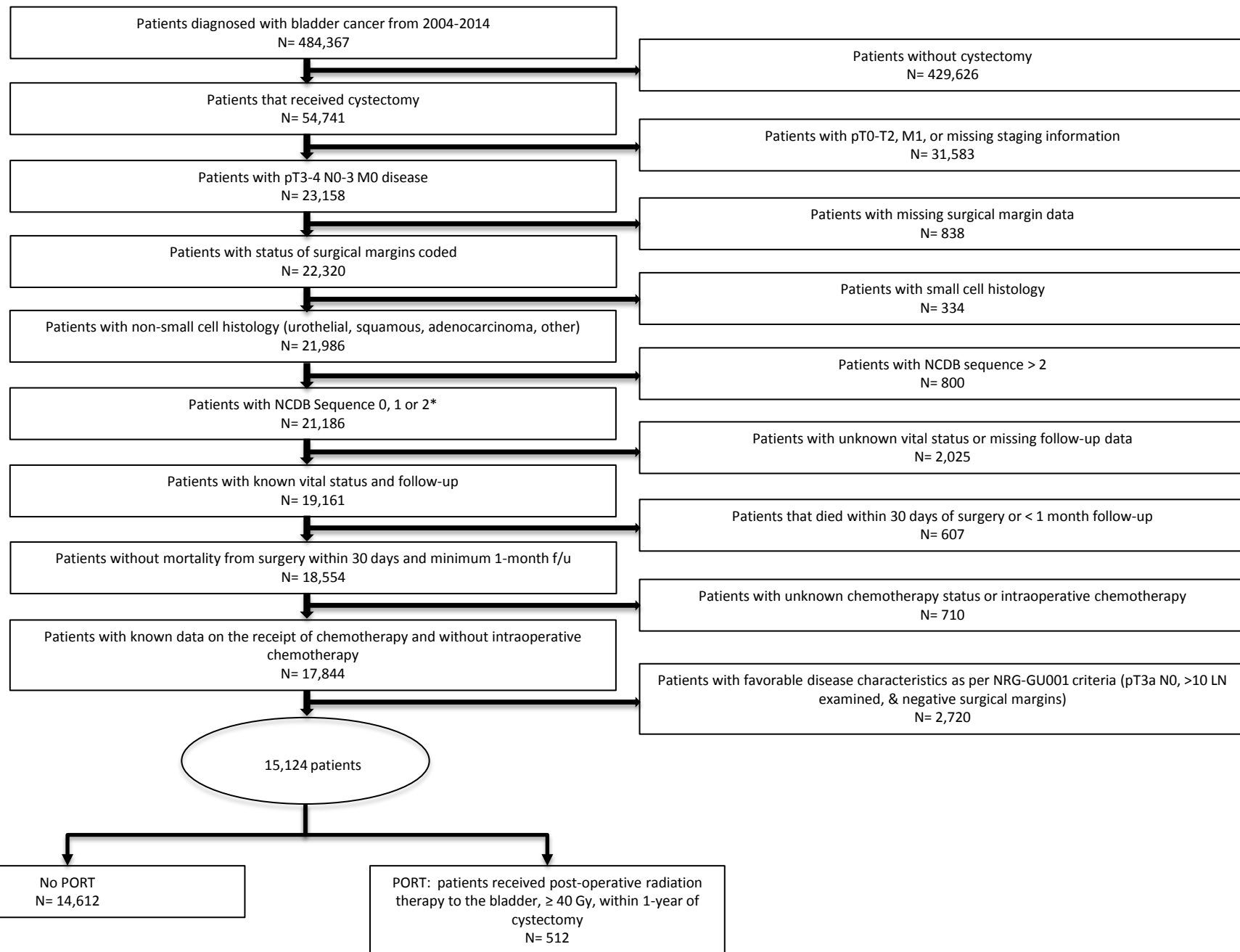

Supplement: Supplementary file 1 [file CAM4-8-3698-s001.pdf]

Supplemental Figure 2.

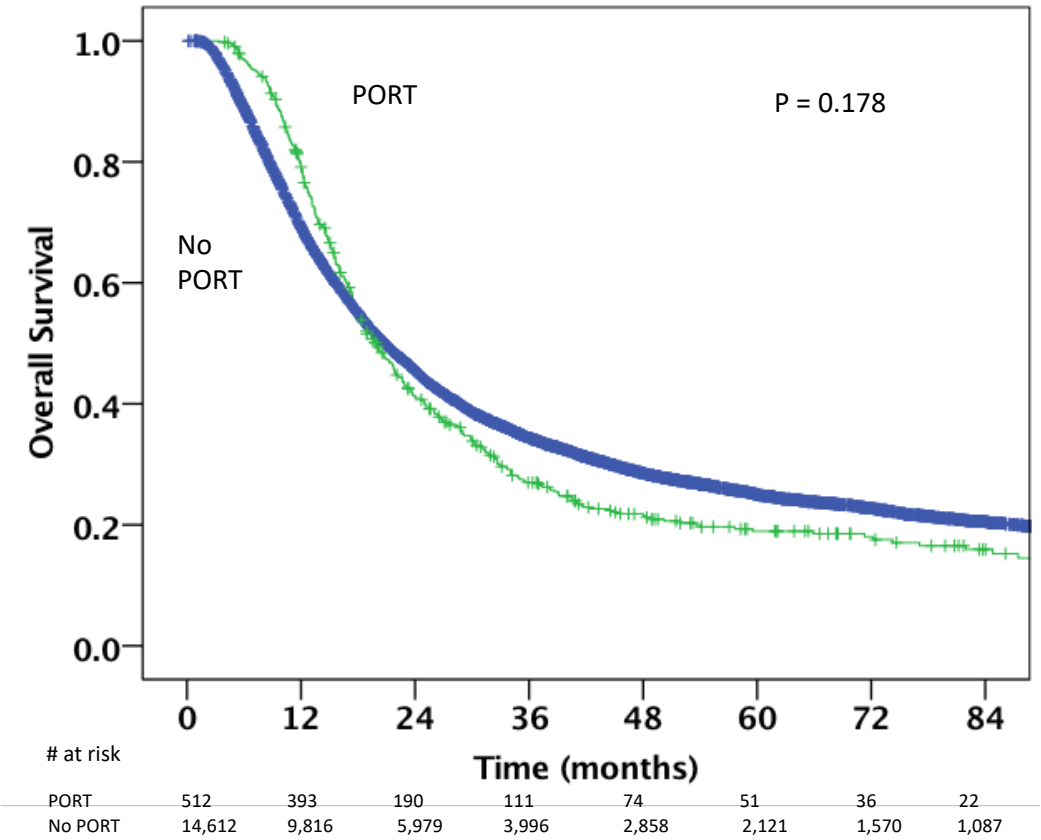

Supplement: Supplementary file 2 [file CAM4-8-3698-s002.pdf]

Supplemental Figure 3.  
(a)

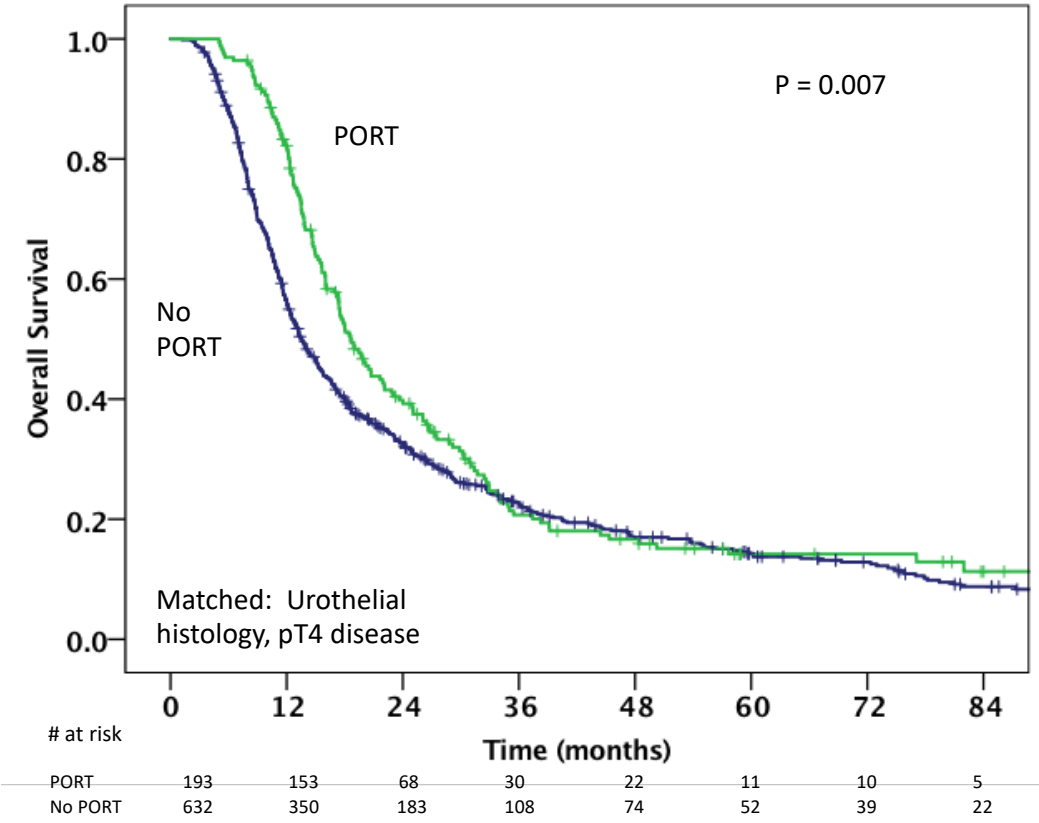

(b)

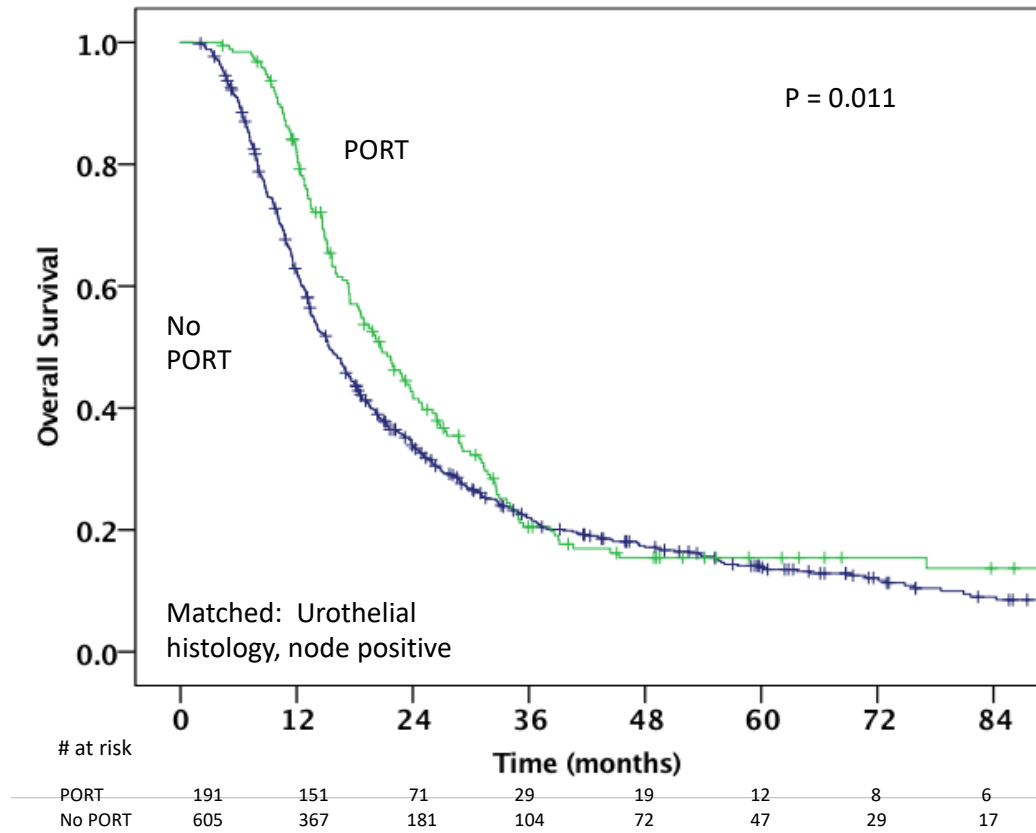

(c)

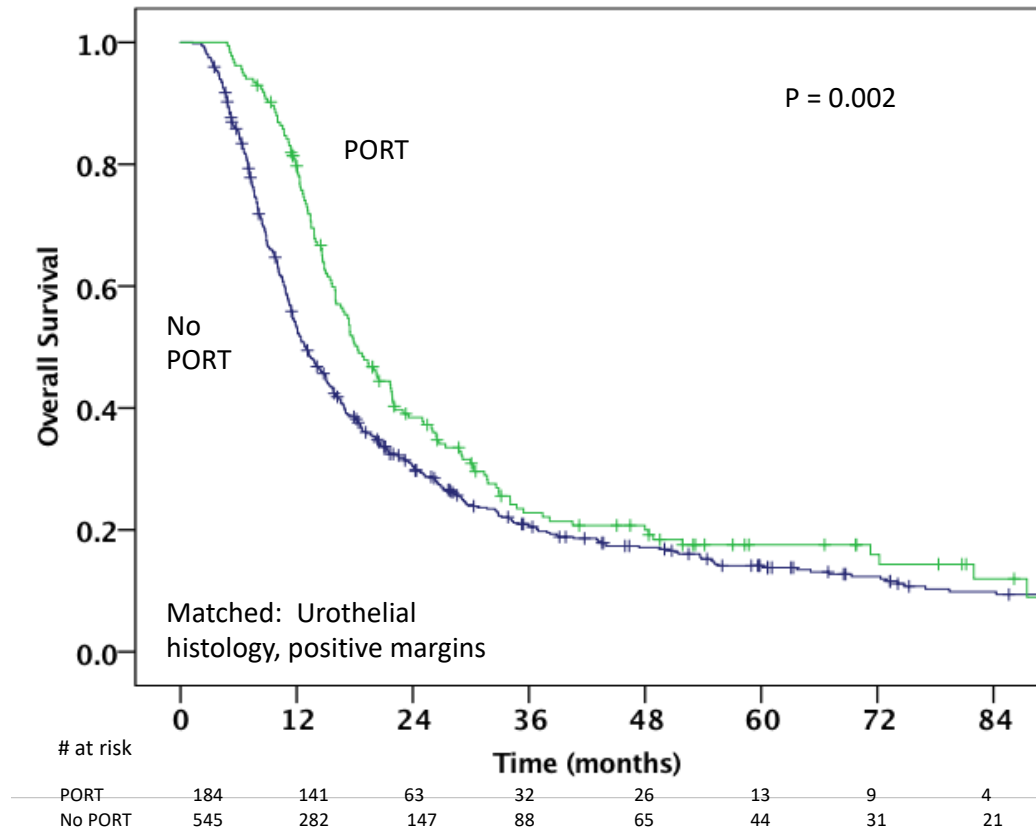

(d)

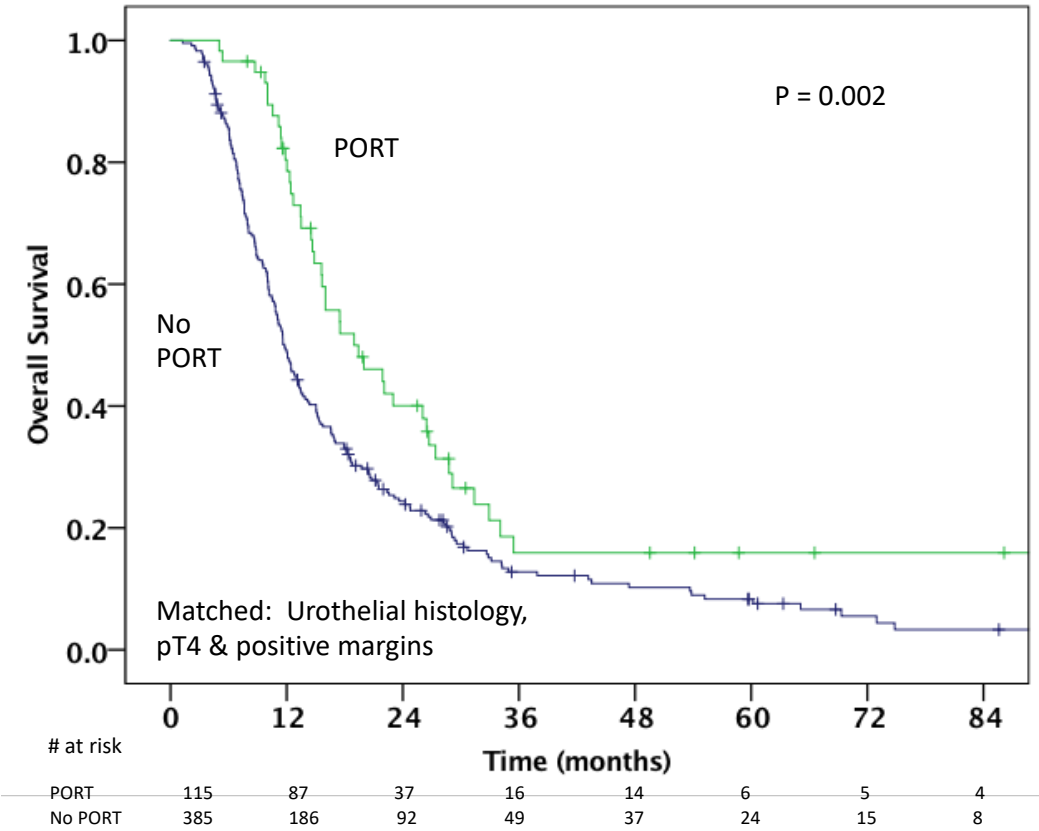

Supplement: Supplementary file 3 [file CAM4-8-3698-s003.pdf]
