# Supplementary material for: Effectiveness of postoperative radiotherapy after radical cystectomy for locally advanced bladder cancer
Source: Cancer Med. 2019 May 22;8(8):3698–709. doi: 10.1002/cam4.2102 (PMC6639450; doi:10.1002/cam4.2102)
Supplement: Supplementary file 4 [file CAM4-8-3698-s004.docx]

**Supplemental Table 1.** Demographics and clinical characteristics of propensity matched cohort

Number of Patients

**No PORT** **PORT**  P-value

N 1,396 (75.1%) 462 (24.9%) **---**

Age, years 0.377

Mean 65.9 64.8

Median 66 66

St. Dev 10.9 10.8

Sex Male 900 (64.5%) 289 (62.6%) 0.468

Female 496 (35.5%) 173 (37.4%)

Race

White 1,227 (87.9%) 410 (88.7%) 0.679

Other 169 (12.1%) 52 (11.3%)

Charlson Deyo Comorbidity:

0 998 (71.5%) 333 (72.1%) 0.898

1 297 (21.3%) 94 (20.3%)

≥2 101 (7.2%) 35 (7.6%)

Facility Type

Academic/Research Program 697 (49.9%) 143 (31.0%) **<0.0001**

Other 683 (48.9%) 310 (67.1%)

Unknown 16 (1.1%) 9 (1.9%)

Insurance Status

Private 467 (33.5%) 163 (35.3%) 0.496

Other 929 (66.5%) 299 (64.7%)

Histology

Urothelial 1,103 (79.0%) 357 (77.3%) 0.741

Squamous 164 (11.7%) 59 (12.8%)

Adeno 45 (3.2% ) 19 (4.1%)

Other (excluding small cell/lymphoma) 84 (6.0%) 27 (5.8%)

Pathologic T-stage

T3 591 (42.3%) 205 (44.4%) 0.448

T4 805 (57.7%) 257 (55.6%)

Positive Lymph Nodes

No 657 (47.1%) 228 (49.4%) 0.420

Yes 739 (52.9%) 234 (50.6%)

Regional Lymph Nodes Examined 0.985

Mean 10.4 10.5

Median 8 8

St. Dev 9.6 10.4

Positive surgical margins

No 700 (50.1%) 226 (48.9%) 0.668

Yes 696 (49.9%) 236 (51.1%)

Adjuvant chemotherapy

No 397 (28.4%) 115 (24.9%) 0.149

Yes 999 (71.6%) 347 (75.1%)
